# Unilateral facial injection of Botulinum neurotoxin A attenuates bilateral trigeminal neuropathic pain and anxiety-like behaviors through inhibition of TLR2-mediated neuroinflammation in mice

**DOI:** 10.1186/s10194-021-01254-2

**Published:** 2021-05-17

**Authors:** Wei-Jia Chen, Jing-Qi Niu, Yi-Ting Chen, Wen-Jing Deng, Ying-Ying Xu, Jing Liu, Wei-Feng Luo, Tong Liu

**Affiliations:** 1 Department of Neurology, The Second Affiliated Hospital of Soochow University, 1055 Sanxiang Road, Suzhou, 215004 China; 2 The First Affiliated Hospital of USTC, Division of Life Sciences and Medicine, University of Science and Technology of China, Hefei, 230001 Anhui China; 3 Changzhou Hygiene Vocational Technology College, Changzhou, 213002 China; 4 Jiangsu Key Laboratory of Translational Research and Therapy for Neuro-Psychiatric-Diseases, Soochow University, Suzhou, 215021 China; 5 Institute of Pain Medicine and Special Environmental Medicine, Nantong University, Nantong, 226019 China; 6 College of Life Sciences, Yanan University, Yanan, 716000 China; 7 Suzhou Key Laboratory of Intelligent Medicine and Equipment, Suzhou, 215123 China

**Keywords:** Trigeminal neuralgia, Anxiety, Microglia, Innate immunity, Toll-like receptor 2 (TLR2), Botulinum toxin type A

## Abstract

**Objectives:**

In this study, we investigated the possible analgesic effects of Botulinum toxin type A (BoNT/A) on trigeminal neuralgia (TN). A modified TN mouse model was established by chronic constriction injury of the distal infraorbital nerve (dIoN-CCI) in mice, and the possible roles of microglia toll-like receptor 2 (TLR2) and neuroinflammation was investigated.

**Methods:**

Male C57BL/6 mice were divided into 3 groups, including sham group, vehicle-treated TN group and BoNT/A-treated TN group. Bilateral mechanical pain hypersensitivity, anxiety-like and depressive-like behaviors were evaluated by using von Frey test, open field, elevated plus-maze testing, and forced swimming test in mice, respectively. The mRNA or protein expression levels of toll-like receptors (TLRs), glia activation markers and proinflammatory factors in the trigeminal nucleus caudalis (TNC) were tested by RT-qPCR, immunofluorescence and Western blotting. We also tested the pain behaviors of TN in *Tlr2*^*−/−*^ mice.

**Results:**

We found that unilateral subcutaneous injection of BoNT/A into the whisker pad on the ipsilateral side of dIoN-CCI mice significantly attenuated bilateral mechanical pain hypersensitivity and anxiety-like behaviors induced by dIoN-CCI surgery in mice. The dIoN-CCI surgery significantly up-regulated the expression of TLR2, MyD88, CD11b (a microglia marker), IL-1β, TNF-α and IL-6 in the ipsilateral TNC in mice, and BoNT/A injection significantly inhibited the expression of these factors. Immunostaining results confirmed that BoNT/A injection significantly inhibited the microglia activation in the ipsilateral TNC in dIoN-CCI mice. TLR2 deficiency also alleviated bilateral mechanical pain hypersensitivity and the up-regulation of MyD88 expression in the TNC of dIoN-CCI mice.

**Conclusion:**

These results indicate that unilateral injection of BoNT/A attenuated bilateral mechanical pain hypersensitivity and anxiety-like behaviors in dIoN-CCI mice, and the analgesic effects of BoNT/A may be associated with the inhibition of TLR2-mediated neuroinflammation in the TNC.

## Background

Trigeminal neuralgia (TN) is possible one of the most severe pain suffered by human [1]. The onset of the disease is usually between 50 and 70 years old. Clinical manifestation of TN often presents as a severe pain like a cut, stab, or electric shock [1]. Current treatments for TN include pharmacological and surgical treatments. Of note, most TN patients are resistant to opioids (such as morphine), which brings a big challenge for the management of TN. Clinically, antiepileptic drugs are often used medications, include carbamazepine, oxcarbazepine, and gabapentin [2]. Along with the TN disease progression, TN patients require higher doses of analgesics to control pain, which leads to more unwanted side effects [2]. For the case of refractory TN, surgical procedures may be a considered strategy, including microvascular decompression and gamma knife surgery [3]. However, abovementioned surgical procedures may have some risks, including ataxia, brainstem injury, and cranial nerve palsy [3]. Therefore, TN patients are in urgent need of a new treatment without side effects.

Under chronic pain conditions, bilateral mechanical pain hypersensitivity in response to unilateral injury is also well-known as mirror image pain [4]. The mechanisms underlying mirror image pain are largely unclear yet. As previously reported, nerve damage increased activity in the contralateral S1 cortex associated with chronic nociception [4]. Previous work demonstrated that early administration of agents that inhibit satellite glial activation or reduce its production of nerve growth factor (NGF) can be effective in relieving mirror image pain induce by spinal nerve ligation (SNL) in rats [4, 5]. However, the detailed mechanisms underlying mirror image pain are still elusive and effective treatments are not available clinically yet.

Botulinum toxins are exotoxins produced by the *Clostridium botulinum*, a gram-positive anaerobic bacillus, during the reproduction process [6]. Botulinum toxin type A (BoNT/A) produces muscle relaxation by inhibiting the release of acetylcholine at the neuromuscular junction, which prevents the downward neurotransmission of nerve impulses and achieves the effect of denervation, resulting in muscle relaxation [6]. Nowadays, BoNT/A is the widely used in clinical practice. In 2002, Micheli reported the first case in which BoNT/A was used to treat TN with significant therapeutic benefits [7]. Our previous study demonstrated that BoNT/A injection attenuates depression-like behavior induced by chronic stress in mice, possible via upregulation of the expression of BDNF in the hippocampus [8]. Depression and anxiety are often observed in patients with chronic pain, with a prevalence of 30% to 50% [9]. Recent studies have shown that neuropathic pain causes anxiety and depression-like behaviour in mice [9]. However, the mechanisms underlying analgesic, anti-depressant or anxiolytic of BoNT/A therapy is still largely unclear.

Injury-induced synaptic plasticity in the pain circuits of the central nervous system (including spinal cord and brain), also known as central sensitization, is responsible for the maintenance of persistent pain and/or widespread pain beyond the initial site of injury of the body [10]. Glia (such as microglia and astrocyte) activation has been demonstrated to be critical for the development of central sensitization, which contributes to the pathogenesis of neuropathic pain [10]. A key aspect of microglia responding to nerve injury is the proliferation (microgliosis) and activation of microglia in the central nervous system (CNS), including the spinal cord. Microglia activation caused by peripheral nerve injury is accompanied by the development of pain hypersensitivity and blocking microglia activation can reduce pain behaviors in animal models. Activated microglia can release pro-inflammatory mediators, such as tumor necrosis factor-α (TNF-α), interleukin-1β (IL-1β), IL-6, brain-derived neurotrophic factor (BDNF) and prostaglandin E2 (PGE2), which promote the initiation and maintenance of neuropathic pain. However, the inhibitory effect of BoNT/A on microglia activation after nerve injury and the detailed mechanisms has not been investigated [11].

Toll-like receptors (TLRs) are normally expressed in immune and glial cells to regulate innate and adaptive immunity [12, 13]. TLR2 gene expression is known to be used as a reliable marker of activated microglia in vivo, but its detailed role in microgliosis remains unknown [14]. Previous studies have also suggested that TLR2 is involved in the pathogenesis of neuropathic pain models. However, the role of TLR2 in TN remains unclear. In 2015, Yun Jeong Kim et al. suggested that in macrophages, BoNT/A may act on TLR2 to cause a decrease in mitogen-activated protein kinase (MAPK) signaling pathway, which caused a decrease in the expression of several pro-inflammatory mediators [15]. Therefore, the purpose of this study was to test whether peripheral (facial) injection of BoNT/A could relieve TN and to further explore possible molecular mechanisms. We used chronic constriction injury (CCI) of the distal infraorbital nerve (dIoN) to establish a modified TN model according to previous report [16]. Then, we explored the analgesic role of BoNT/A in TN and its mechanism of action in TNC by using behavioral and molecular biological approaches. Hopefully, we expect to provide an effective BoNT/A treatment for the management of TN.

## Methods

### Animals

Six to eight weeks old male C57BL/6 J mice (weighing over 20 g) were provided by experimental animal center of Soochow University. We randomly assigned male C57BL/6 J mice of matched age to the sham-operated group, dIoN-CCI + vehicle group and dIoN-CCI + BoNT/A treated group. The group of mice during testing was blinded to the experimenter of the behavioral test. Male TLR2 knockout mice (*Tlr2*^−/−^; stock#005846) were purchased from the Jackson Laboratory and raised at Soochow University Laboratory Animal Center. They were housed at five to six per cage, habituated to the colony room for a week before the experiments were performed, and kept under a 12 h light/12 h night cycle. The rearing environment was maintained at a constant room temperature of 22 °C and 60%–80% humidity. In addition, water and food were freely available. All procedures were approved by the Animal Care and Use Committee of Soochow University. The number of mice used in each experiments are shown in Table 1. This animal study followed the ARRIVE guidelines [17].

**Table 1:** Animal numbers in each group

Experimental group	Behavioral tests	RT-qPCR	WB	IF	Total
Sham	12^a^	4	4	4	12
dIoN-CCI	18^a^	4	10	4	18
dIoN-CCI + BoNT/A	12^a^	4	4	4	12
*Tlr2*^−/−^ dIoN-CCI	6^a^	–	6	–	6
Total					48

^a^Indicates shared with other experiments not counted in total

### Mouse model of TN

The chronic constriction injury (CCI) of the distal infraorbital nerve (dIoN) was established following the previous research by Weihua Ding et al. [16]. Without damaging the whiskers, gently shave the facial surface between the mouse’s eyes and the whisker pad. Made a 0.5 cm incision parallel to the midline, starting from the caudal end of the third row of whisker lines and then toward the ipsilateral orbit. The superficial fascia was bluntly separated and the main trunk of the infraorbital nerve (IoN) outside the orbital cavity was exposed in its distal segment. Two chromic catgut ligatures (6–0) were tied loosely to the distal portion of the IoN (1 mm distance). To ensure proper constriction of the IoN, the ligatures were applied in such a way that the diameter of the IoN was reduced and superficial vascular circulation was retarded but not severed. Skin incisions were closed with polyester sutures (6–0). The sham-operated group of mice underwent the same surgical procedure, including skin incision and IoN nerve exposure, except that the actual nerve was ligated.

### Drugs and administration

The following reagents were obtained from the sources indicated: BoNT/A (GMP nos S10970037) was obtained from the Lanzhou Institute of Biological Products, Lanzhou, China. Each vial contains 100 U of purified C. Botulinum type A (BoNT/A) neurotoxin complex. The toxin was stored at − 80 °C in 0.9% NaCl. All other chemicals used in the study were of analytical grade and obtained from commercial sources. Two weeks after dIoN-CCI surgery, 0.18 U BoNT/A was injected subcutaneously (s.c.) into the whisker pad on the ipsilateral side of the dIoN-CCI mice.

### Mechanical pain test

Von Frey filaments were used to measure mechanical pain in the bilateral whisker pad of mice. We performed pilot experiments to measure mechanical pain by the “up-down method” and found that mice had an obvious positive pain response at 0.008 g. The intensity of the stimulus filaments was set at 0.008 g and 0.02 g. Mice with positive pain responses were tested as described by Weihua Ding et al. [16]. Each mouse was placed in a small transparent plastic cage for 1 h to acclimatize to the experimental environment, and the experimenter was blinded to the group assignment of the mice. The number of positive reactions produced by both sides of the mice was counted. Each stimulus was given 10 times on each side and repeated three times. The final mouse mechanical pain was calculated as a percentage of positive responses in the total number of stimuli. We tested the basal mechanical pain positive response rate followed by dIoN-CCI 1 week later and weekly thereafter. To study the analgesic effect of BoNT/A, after BoNT/A injection, mechanical pain testing was performed at 1 h, 1 day, after that, the test was conducted every 4 days.

### Open field test

To evaluate the behavior of three groups of mice in the open field, mice were individually placed into a 40*40 cm center area of a brightly illuminated open field arena. The bottom was divided into a 4*4 grid, 16 equal sized (10 cm*10 cm) squares. Mice were allowed to explore the stage undisturbed for 10 min. Video tracking software was used to count the time the mice entered the central bright area (the middle four squares). And the excretion rate of the three groups of mice during the test was counted.

### Elevated plus-maze testing

Mice were taken individually to the chamber 1 h before the start of the trial. The elevated plus maze device was made of white opaque plastic plates and an iron trestle table. It consisted of two enclosed arms and two open arms (36 cm*6 cm) fixed to the central platform. There was a 6 cm*6 cm open section in the center of the maze. The two enclosed arms were surrounded on three sides by 15 cm high walls, and the two open arms had no walls. The maze was elevated to a height of 60 cm [18]. When tested, the mice were placed individually in the center of the maze with their heads facing an open arm (the same for all mice). Before the next mouse was tested, the instrument was wiped clean with an absorbent cotton wool. Each mouse was videotaped for 5 min. During the 5 min test period, the duration and number of entries into the open and closed arms were counted separately (mice with all four limbs entered were included in the count). These measures enabled the calculation of the duration and number of times the mice entered the open or the closed arms. The number of times mice entered the open arms and the duration in the open arms were calculated as a percentage of the total number of entries (sum of open and closed arms entries) and total time (sum of duration in both arms). The anxiolytic effect was represented by an increase in the percentage of the number of times the mouse entered the open arms and the percentage of the time spent in the open arms, but the total number of entries and the total time spent in the arms remained unchanged. On the contrary, the anxiogenic effect was reflected that both percentages decreased without affecting the total number of entries and the duration in both arms.

### Forced swimming test

Forced swimming tests were performed on mice before surgery, during the second week after surgery and on day 5 after BoNT/A injection. Mice were placed in a glass bucket (18 cm in diameter and 30 cm in height) filled with 10 cm high water at a temperature of 23°C ± 1°C. Mice were left in the water for 6 minutes to measure the immobility time of each mouse. Immobility was defined as the absence of any active movement of the mice, except for the small movements required to keep them afloat on the water surface.

### Western blot analysis

On day 5 after BoNT/A injection, the mice were under deep anesthesia with 4% chloral hydrate (10 ml/kg, i.p.), intracardiac perfusion was performed with 0.9% saline. The bilateral TNC were quickly removed and then frozen in liquid nitrogen. For further western blot analysis, samples were homogenized in protein lysis buffer containing phosphatase inhibitor and protease inhibitor proteolytic buffer for total protein extraction assays. Protein concentrations were determined by Pierce BCA protein assay (Thermo). After concentration determination and denaturation, protein samples (30 μg) were separated on SDS-PAGE gels and electrotransferred onto nitrocellulose membranes. After sealing with 5% fat-free milk, PVDF membranes were incubated with primary polyclonal anti-MyD88 (rabbit, 1:1000; abcam). For loading control, PVDF membranes were incubated with β-tubulin antibody (mouse, 1:2000, Vazyme, Nanjing, China) at 4 °C overnight. The washed blots were incubated with horseradish peroxidase-conjugated goat anti-rabbit and goat anti-mouse IgG secondary antibodies (1:2000, Vazyme, Nanjing, China) for 1 h at room temperature. The washed protein bands were developed using ultrasensitive ECL chemiluminescence kit (NCMECL Ultra) and analyzed for grayscale values as indicated. The ratio between MyD88 and β-tubulin was calculated and then normalized to the control measurements. Data from four mice were used for statistical analysis.

### Quantitative real-time polymerase chain reaction

Total RNA was isolated from frozen tissues by guanidinium isothiocyanate-phenol extraction and quantified by measuring absorbance at 260 nm and 280 nm. 1 μg of total RNA was used for reverse transcription. TLRs, inflammatory factors and cell marker mRNA of TNC were quantified by qPCR (prism 7500; Applied Biosystems, Foster, California). The expression of the relevant mRNAs were normalized by the expression of glyceraldehyde-3 phosphate dehydrogenase mRNA. The following table shows the primer sequences used for qPCR [19]. Data from four mice were used for statistical analysis. The primer sequences used in the RT-qPCR are shown in Table 2.

**Table 2:** The primers used in RT-qPCR

	Forward primers	Reverse primers	Accession No.
Gapdh	TTGATGGCAACAATCTCCAC	CGTCCCGTAGACAAAATGGT	NM_001001303
Tlr1	ATGTGAGCTGAGGGACTTTG	GGATAGTGGAGACATGTGGAAG	NM_030682
Tlr2	ACCAAGATCCAGAAGAGCCA	CATCACCGGTCAGAAAACAA	NM_011905
Tlr3	GCGTTGCGAAGTGAAGAACT	TTCAAGAGGAGGGCGAATAA	NM_126166
Tlr4	TTCAGAACTTCAGTGGCTGG	TGTTAGTCCAGAGAAACTTCCTG	NM_021297
Tlr5	GCAGGATCATGGCATGTCAAC	ATCTGGGTGAGGTTACAGCCT	NM_016928
Tlr6	CCAAGAACAAAAGCCCTGAG	TGTTTTGCAACCGATTGTGT	NM_011604
Tlr7	GATGTCCTTGGCTCCCTTC	TTTGTCTCTTCCGTGTCCAC	NM_133211
Tlr8	CGTTTTACCTTCCTTTGTCTATAGAAC	CGTCACAAGGATAGCTTCTGG	NM_133212
Tlr9	AACCGCCACTTCTATAACCAG	GTAAGACAGAGCAAGGCAGG	NM_031178
Tlr11	CAGGCTGGGATTGCTCATC	CCAGTCAAGGTAAGGCTCAC	NM_205819
Tlr12	GCTCTGATTCCTCTGGTGTAG	AGAATGTGAAATAGCGGGAGAC	NM_205823
Tlr13	GGAGCGCCTTGATCTAACTAACA	TCAGGTGGGTCAGAGAAACCA	NM_205820
IL-1β	AGAGCATCCAGCTTCAAATCTC	CAGTTGTCTAATGGGAACGTCA	NM_008361.4
TNF-α	AGCCGATGGGTTGTACCTTG	TTGGGCAGATTGACCTCAGC	NM_001278601.1
IL-6	TCAGGAAATTTGCCTATTGAAAA	CCAGCTTATCTGTTAGGAGAGCA	NM_031168.2
c-Fos	GACAGCCTTTCCTACTACCA	CATCTTATTCCTTTCCCTTCG	XM_021201871.2
F4/80	ATGGACAAACCAACTTTCAAGGC	GCAGACTGAGTTAGGACCACAA	XM_036160297.1
GFAP	GAATCGCTGGAGGAGGAGAT	GCCACTGCCTCGTATTGAGT	XM_030245571.2
CD11b	GACATGGACGCTGATGGCAATAC	CGAGGCAAGGGACACACTGAC	NM_008401.2

## Immunofluorescence (IF)

On day 5 after BoNT/A injection, the mice were completely anesthetized with 4% chloral hydrate (10 ml/kg, i.p.) and then perfused with sterile saline throughout the body, followed by fixation with 4% paraformaldehyde. After fixation, the segment where the mouse TNC was located was taken and the same fixative was left overnight. After sucrose gradient dehydration, the tissues were embedded with OCT tissue embedding agent and frozen at − 80 °C in the refrigerator. The embedded tissues were sectioned (20 μm thickness) and processed on a cryostat microtome (CM 1950; Leica Microsystems, Wetzlar, Germany). Immunohistochemistry was performed as we described previously [20]. Briefly, sections were incubated with 5% goat serum and incubated overnight at 4 °C with primary anti IBA1 antibody (rabbit, 1:500, Wako) and anti TLR2 antibody (mouse, 1:100, abcam). Then after washing away the first antibody, sections were incubated with FITC and Cy3 conjugated secondary antibodies for 1 h at room temperature. Immunostained sections were examined under a ZEISS fluorescence microscope (Carl Zeiss OPMI Pentero, Germany), images were taken and the sections were examined with NIH ImageJ software (NIH, Bethesda, MD) for analysis.

### Statistical analysis

The data was analyzed using Graph Prism 6 (Graph Pad, La Jolla, CA). Shapiro-Wilk test was used to test normality of the data. All values were presented as mean ± SEM. Unpaired Student’s t-test was used to compare two groups. Two-Way Repeated Measures ANOVA with *post-hoc* Bonferroni test was performed for multiple comparisons. Differences were considered statistically significant at *P* < 0.05.

## Results

### A modified TN model was established by chronic constriction injury (CCI) of the unilateral distal infraorbital nerve (dIoN) in mice

**Fig. 1:**
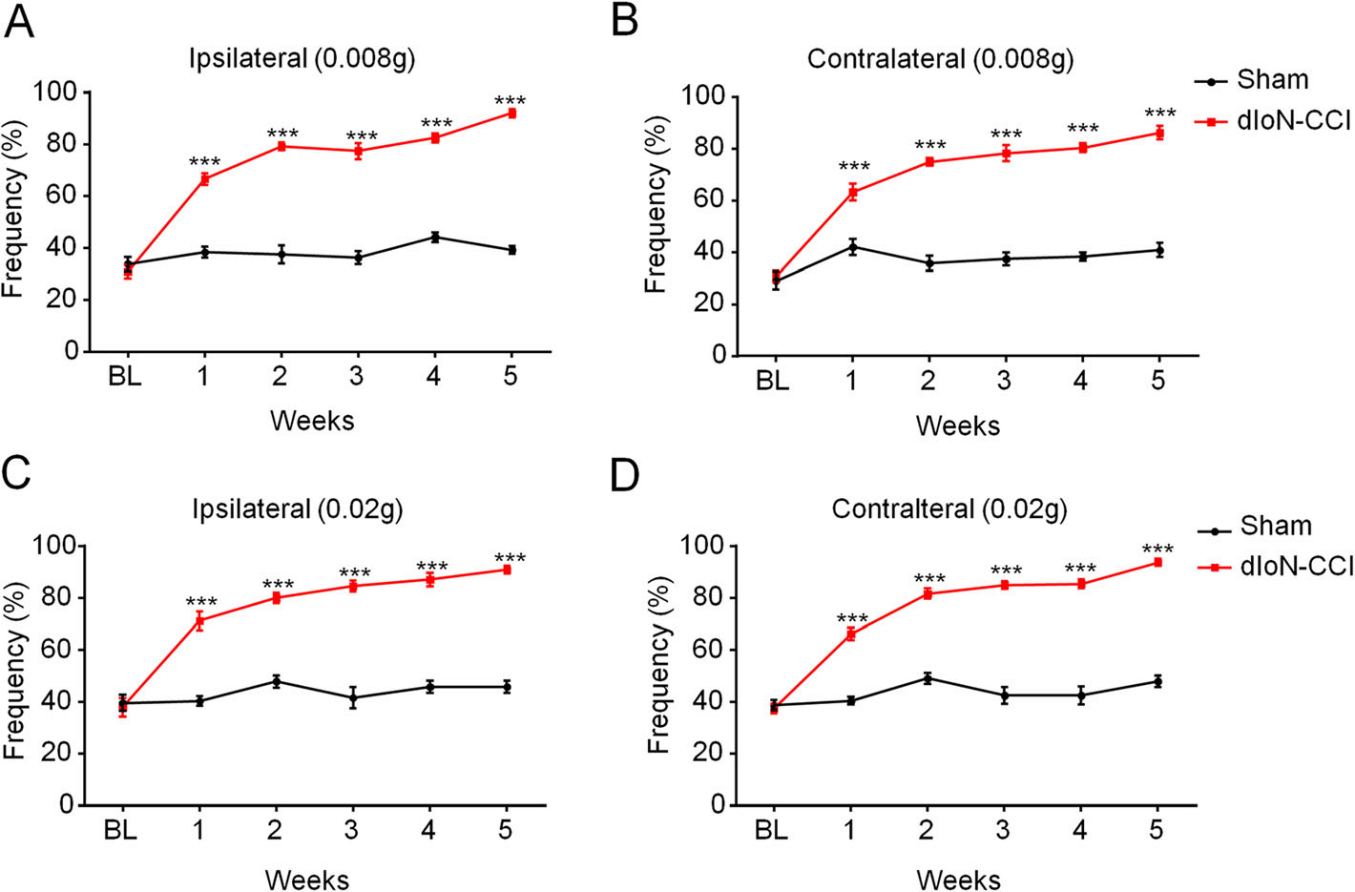
Bilateral mechanical pain hypersensitivity during 5 weeks following dIoN-CCI surgery in mice. **a**-**b** At 0.008 g stimulus intensity, mechanical pain hypersensitivity in dIoN-CCI mice was observed in the ipsilateral (**a**) and contralateral side (**b**) to dIoN-CCI surgery compared with that of sham mice. **c**-**d** At 0.02 g stimulus intensity, mechanical pain hypersensitivity in dIoN-CCI mice was observed in the ipsilateral (**c**) and contralateral side (**d**) to dIoN-CCI surgery compared with that of sham mice. Data was expressed as Mean ± SEM. (*n* = 8 each group; ^***^ *P* < 0.001 compared with the sham group, two-way ANOVA with *post-hoc* Bonferroni test). dIoN-CCI, chronic constriction injury of distal infraorbital nerve; BL, baseline

We used von Frey filaments to evaluate mechanical pain sensitivity in the bilateral whisker pad of mice before surgery by measurement of the frequency of positive reactions to the filaments in mice. One week after dIoN-CCI surgery, the bilateral mechanical pain sensitivity in the whisker pad was detected. Compared to that of the sham animals, dIoN-CCI mice showed a significant mechanical pain hypersensitivity on the injured side at 0.008 g mechanical stimulus intensity (F_time(5, 35)_ = 69, *P* < 0.0001; F_surgery(1, 7)_ = 541.3, *P* < 0.0001; F_time × surgery(5, 35)_ = 37.62, *P* < 0.0001; Fig. 1a), and the similar results were also observed on the contralateral side (F_time(5, 35)_ = 58.97, *P* < 0.0001; F_surgery(1, 7)_ = 203.8, *P* < 0.0001; F_time × surgery(5, 35)_ = 27.08, *P* < 0.0001; Fig. 1b). At 0.02 g mechanical stimulation intensity, the frequency of positive responses to filaments had the same trend as the 0.008 g stimulation intensity (ipsilateral: F_time(5, 35)_ = 36.46, *P* < 0.0001; F_surgery(1, 7)_ = 453.6, *P* < 0.0001; F_time × surgery(5,35)_ = 21.78, *P* < 0.0001; Fig. 1c; contralateral: F_time(5, 35)_ = 83.26, *P* < 0.0001; F_surgery(1,7)_ = 468, *P* < 0.0001; F_time × surgery(5, 35)_ = 34.68, *P* < 0.0001; Fig. 1d). Thus, these data indicated that a modified mouse model of trigeminal neuralgia (TN) was successfully established and we found that unilateral ligation of the distal infraorbital nerve caused bilateral mechanical pain hypersensitivity in the whisker pad lasting for at least 5 weeks.

### Ipsilateral facial subcutaneous injection of BoNT/A attenuated bilateral mechanical pain hypersensitivity caused by dIoN-CCI surgery in mice

**Fig. 2:**
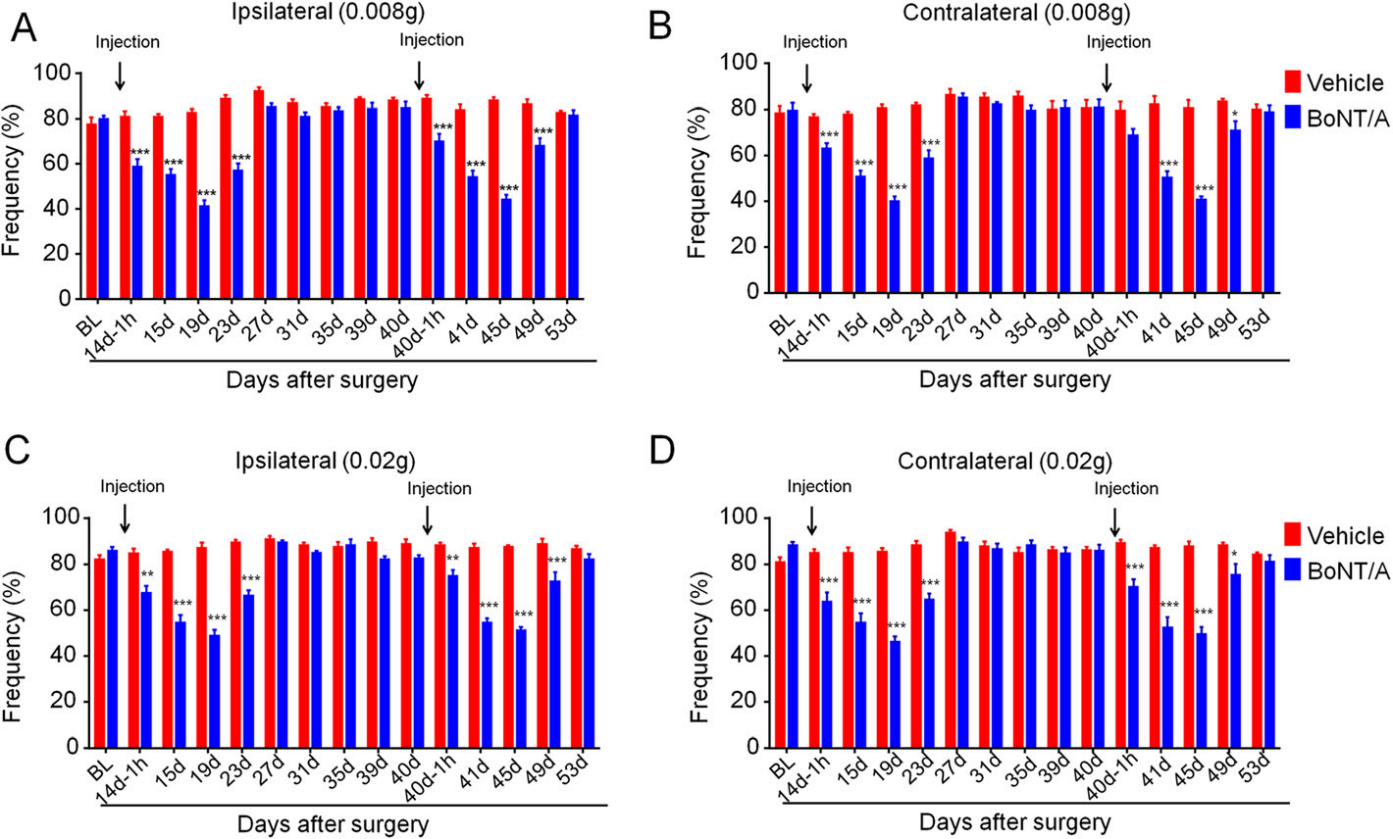
Unilateral facial injection of BoNT/A attenuated bilateral mechanical pain hypersensitivity induced by dIoN-CCI surgery in mice. Black arrow represented the time of BoNT/A injection in mice. **a**-**b** At 0.008 g von Frey stimulation intensity, facial injection of BoNT/A significantly attenuated ipsilateral (**a**) and contralateral (**b**) mechanical pain hypersensitivity induced by dIoN-CCI surgery in mice, which lasted approximately 9 days, compared to the vehicle group. After 2 weeks, a second injections of BoNT/A showed the similar analgesic effect. **c**-**d** At 0.02 g von Frey stimulation intensity, facial injection of BoNT/A significantly attenuated ipsilateral (**a**) and contralateral (**b**) mechanical pain hypersensitivity induced by dIoN-CCI surgery in mice, which lasted approximately 9 days, compared to the vehicle group. After 2 weeks, a second injections of BoNT/A showed the similar analgesic effect. Data was expressed as Mean ± SEM. (*n* = 8 each group, ^*^ *P* < 0.05, ^**^P < 0.01, ^***^P < 0.001, two-way ANOVA with *post-hoc* Bonferroni test). dIoN-CCI, chronic constriction injury of distal infraorbital nerve; BoNT/A botulinum toxin A; BL, baseline

After establishment of TN mouse model, dIoN-CCI mice were divided into two groups, TN + vehicle group and TN + BoNT/A group. We subcutaneously injected BoNT/A into the ipsilateral whisker pad, and assessed the analgesic effect of BoNT/A by examining the bilateral mechanical pain hypersensitivity in the whisker pad in dIoN-CCI mice. At 0.008 g mechanical stimulation intensity, the analgesic effect of BoNT/A injection appeared after 1 h injection into the ipsilateral side, and the analgesic effect was still detectable on the fifth day after BoNT/A injection. The analgesic effect of BoNT/A lasted for about 9 days. Two weeks after the analgesic effect had worn off, a second injections of BoNT/A was performed to test the analgesic effects of repeated injection of BoNT/A in mice. The data showed a second facial injection of BoNT/A produced similar analgesic effect in the ipsilateral side of dIoN-CCI surgery in mice (F_time(14, 98)_ = 28.3, *P* < 0.0001; F_treatment(1, 7)_ = 134.3, *P* < 0.0001; F_time × treatment(14, 98)_ = 27.11, *P* < 0.0001; Fig. 2a). The analgesic effect was also evident on the contralateral side of dIoN-CCI surgery in mice (F_time(14, 98)_ = 26.08, *P* < 0.0001; F_treatment(1,7)_ = 49.41, *P* < 0.0001; F_time × treatment(14, 98)_ = 15.43, *P* < 0.0001; Fig. 2b). The similar analgesic effect at 0.02 g mechanical stimulation intensity had been observed on the bilateral sides of dIoN-CCI surgery in mice (ipsilateral: F_time(14, 98)_ = 25.59, *P* < 0.0001; F_treatment(1,7)_ = 213.4, *P* < 0.0001; F_time × treatment(14, 98)_ = 24.26, *P* < 0.0001; Fig. 2c; contralateral: F_time(14, 98)_ = 23.95, *P* < 0.0001; F_treatment(1,7)_ = 55.33, *P* < 0.0001; F_time × treatment(14, 98)_ = 27.75, *P* < 0.0001; Fig. 2d). Thus, these results suggested that unilateral subcutaneous injection of BoNT/A is an effective and reproducible treatment for the relief of trigeminal neuropathic pain, including mirror image pain in mice.

### Facial injection BoNT/A relieved anxiety-like behavior (but not depression-like behavior) induced by dIoN-CCI surgery in mice

**Fig. 3:**
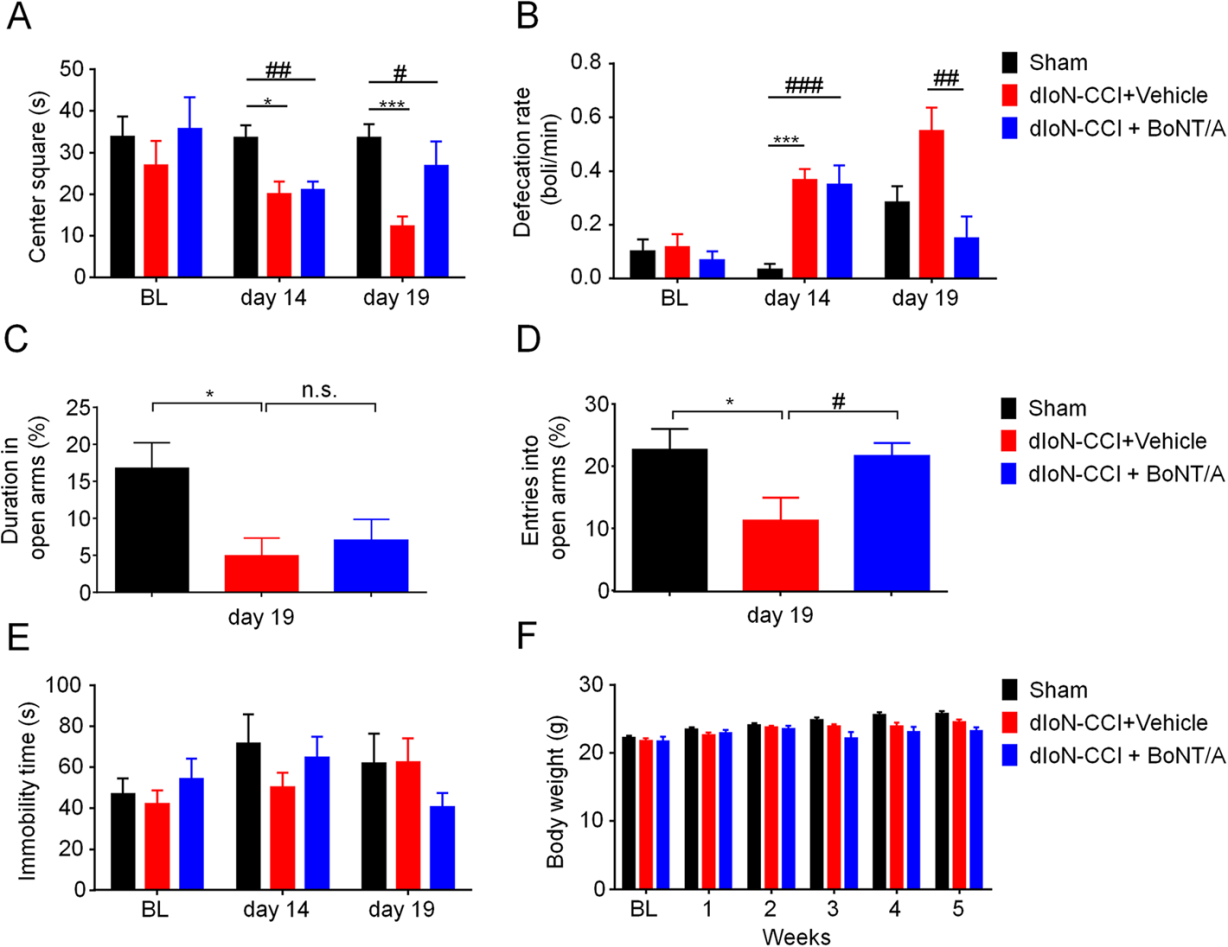
Facial injection of BoNT/A attenuated dIoN-CCI surgery-induced anxiety-like behavior, but not depression-like behavior, in mice. On day 14 following dIoN-CCI surgery, BoNT/A was injected in dIoN-CCI mice. **a** In open field test, on day 5 after BoNT/A injection (day 19 following dIoN-CCI surgery), BoNT/A treatment significantly increase the time in the center area compared with that of vehicle group. **b** On day 5 after BoNT/A injection (day 19 following dIoN-CCI surgery), BoNT/A treatment significantly decrease the excretion rate compared with that of vehicle group. **c**-**d** In an elevated plus maze, on day 5 after BoNT/A injection (day 19 following dIoN-CCI surgery), BoNT/A treatment had no significant effect on the percentage of duration of open arms compared with that of vehicle group (**c**). BoNT/A treatment significantly increased the percentage of entries into the open arm in an elevated plus maze than that of vehicle group (**d**). **e**-**f** There was no significant change in the duration of the immobility time in FST test (**e**), the body weight (**f**) among the three groups during the experiment. Data was expressed as Mean ± SEM. (*n* = 8 each group, n.s., no significance, ^*^ *P* < 0.05, ^***^ *P* < 0.001 compared with the sham group. ^**#**^ *P* < 0.05, ^**##**^ *P* < 0.01, ^**###**^ *P* < 0.001 compared with the dIoN-CCI group, unpaired Student’s t-test). dIoN-CCI, chronic constriction injury of distal infraorbital nerve; BoNT/A, botulinum toxin A; BL, baseline; FST, forced swimming test

On day 14 after dIoN-CCI surgery in mice, we performed the open field test. We found that dIoN-CCI mice took less time to enter the intermediate area than the sham group. On day 5 after BoNT/A injection (day 19 after dIoN-CCI surgery), the BoNT/A-treated dIoN-CCI mice spent more time in the center area compared to the vehicle-treated group (on day 14 before BoNT/A injection: sham vs. dIoN-CCI, t_9_ = 3.145, *P* = 0.0118; dIoN-CCI vs. dIoN-CCI + BoNT/A, t_9_ = 3.368, *P* = 0.0083; on day 5 after BoNT/A injection: sham vs. dIoN-CCI, t_10_ = 5.218, *P* = 0.0004; dIoN-CCI vs. dIoN-CCI + BoNT/A, t_9_ = 2.484, *P* = 0.0348; Fig. 3a). Additionally, on day 14 after dIoN-CCI surgery, dIoN-CCI mice excreted at a higher rate than the sham group. On day 5 after BoNT/A injection (day 19 after dIoN-CCI surgery), the excretion rate of mice was reduced compared to the vehicle-treated group (on day 14 before BoNT/A injection: sham vs. dIoN-CCI + vehicle, t_10_ = 7.071, *P* < 0.0001; dIoN-CCI + vehicle vs. dIoN-CCI + BoNT/A, t_10_ = 4.227, *P* = 0.0018; on day 5 after BoNT/A injection: dIoN-CCI + vehicle vs. dIoN-CCI + BoNT/A, t_10_ = 3.422, *P* = 0.0065; Fig. 3b). Furthermore, we performed the elevated plus maze experiment on day 5 after BoNT/A injection (day 19 after dIoN-CCI surgery), the mice showed a significant decrease in the proportion of duration into the open arms compared to the vehicle-treated group (t_11_ = 2.742; *P* = 0.0192; Fig. 3c). There was a decrease in the percentage of times access to the open arm (sham vs. dIoN-CCI + vehicle, t_10_ = 2.299, *P* = 0.0443; dIoN-CCI + vehicle vs. dIoN-CCI + BoNT/A, t_9_ = 2.320, *P* = 0.0455; Fig. 3d). We also employed forced swimming experiments to study the depression-like behavior in mice. We found no significant differences in the duration of immobility in the tested three groups of mice (Fig. 3e). Additionally, there was also no significant change in the body weight of the three groups of mice during the experiment (Fig. 3f). These results suggested that TN induced anxiety-like behavior (rather than depression-like behavior) in mice, and facial injection of BoNT/A significantly relieved anxiety-like behaviors induced by TN in mice.

### Facial injection of BoNT/A down-regulated the expression of toll-like receptors (TLRs) in the TNC in dIoN-CCI surgery mice

**Fig. 4:**
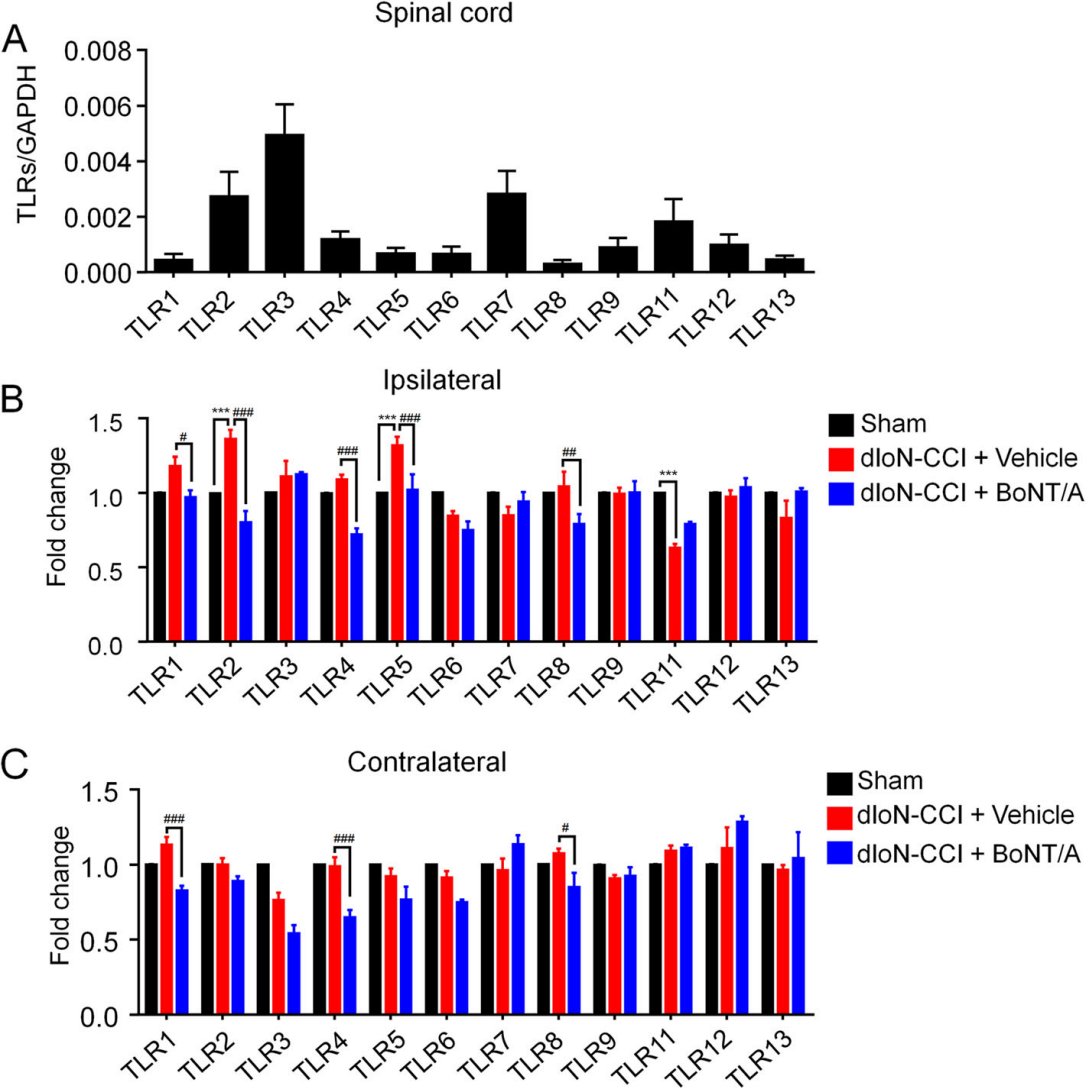
Expression of TLRs in bilateral TNC using RT-qPCR. **a** RT-qPCR analyzed the expression levels of TLRs in wild-type mice. GAPDH was used as internal control (*n* = 6). **b** Relative expression of TLRs in the ipsilateral TNC on day 5 after BoNT/A injection (day 19 following dIoN-CCI surgery) among the three groups. There was an increase in TLR2 and TLR5, a decrease in TLR11 in the dIoN-CCI group compared with the sham group, while there was a decrease in TLR1, TLR2, TLR4, TLR5 and TLR8 expression after BoNT/A injection. **c** Relative expression of TLRs in the contralateral TNC on day 5 after BoNT/A injection (day 19 following dIoN-CCI surgery) among the three groups. The dIoN-CCI and sham group showed no significant change in TLRs expression, while TLR1, TLR4 and TLR8 expression decreased after BoNT/A injection. Data was expressed as Mean ± SEM. (*n* = 4 each group, ^***^ *P* < 0.001 compared with the sham group. ^**#**^ *P* < 0.05, ^**##**^ *P* < 0.01, ^**###**^ *P* < 0.001 compared with the dIoN-CCI group, unpaired Student’s t-test). TLRs, toll-like receptors; TNC, trigeminal nucleus caudalis; dIoN-CCI, chronic constriction injury of distal infraorbital nerve; BoNT/A, botulinum toxin–A; BL, baseline

To investigate the role of TLRs in the pathogenesis of TN, we first examined the expression of TLRs in the TNC in wild-type (WT) male mice. We found that all TLRs were expressed in the TNC with different expression levels in naive mice (Fig. 4a). We then examined the expression of TLRs in the bilateral TNC of the tested three groups. In the ipsilateral TNC, dIoN-CCI surgery caused a significant increase in the mRNA expression of TLR2 and TLR5, a significant decrease in the mRNA expression of TLR11 in mice (sham vs. dIoN-CCI + vehicle, TLR2: t_6_ = 6.307, *P* = 0.0007; TLR5: t_6_ = 7.584, *P* = 0.0003; TLR11: t_6_ = 10.8, *P* < 0.0001). On day 5 after BoNT/A injection (day 19 after dIoN-CCI surgery), BoNT/A-treated mice showed a significant decrease in the mRNA expression of TLR1, TLR2, TLR4, TLR5 and TLR8, compared with vehicle-treated mice (dIoN-CCI + vehicle vs. dIoN-CCI + BoNT/A, TLR1: t_6_ = 3.393, *P* = 0.0146; TLR2: t_6_ = 6.058, *P* = 0.0009; TLR4: t_6_ = 8.721, *P* = 0.0001; TLR5: t_6_ = 6.897, *P* = 0.0005; TLR8: t_6_ = 5.766, *P* = 0.0012; Fig. 4b). On the contralateral side, the dIoN-CCI surgery and sham groups showed no significant change in the mRNA expression of TLRs. However, the expression TLR1, TLR4 and TLR8 were significantly decreased on day 5 after BoNT/A injection (day 19 after dIoN-CCI surgery) compared with vehicle-treated mice (TLR1: t_6_ = 6.614, *P* = 0.0006; TLR4: t_6_ = 6.963, *P* = 0.0004; TLR8: t_6_ = 2.7, *P* = 0.0356; Fig. 4c). Thus, these results suggested that changes of TLRs expression in the TNC were involved in the pathogenesis of TN and BoNT/A treatment significantly down-regulated the mRNA expression of TLRs, particularly TLR2 and TLR5.

### TLR2 deficiency alleviates bilateral mechanical pain hypersensitivity in the dIoN-CCI mice

**Fig. 5:**
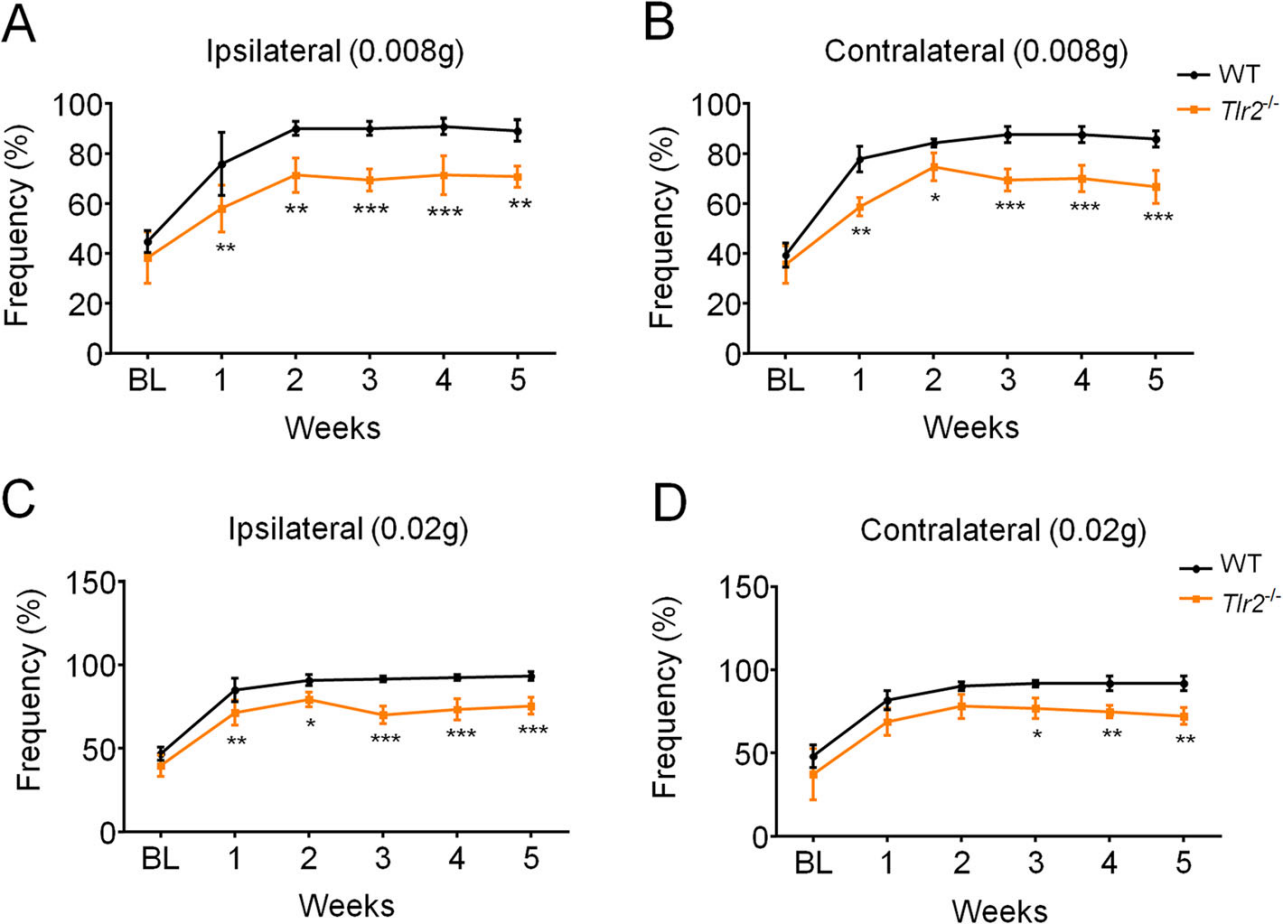
Bilateral mechanical pain hypersensitivity following dIoN-CCI surgery in the wide-type and *Tlr2*^−/−^ mice. **a**-**b** At 0.008 g stimulus intensity, ipsilateral (**a**) and contralateral (**b**) mechanical pain hypersensitivity induced by dIoN-CCI group was significantly inhibited in the *Tlr2*^−/−^ mice compared with that of wild-type mice. **c**-**d** At 0.02 g stimulus intensity, ipsilateral (**c**) and contralateral (**d**) mechanical pain hypersensitivity induced by dIoN-CCI group was significantly inhibited in the *Tlr2*^−/−^ mice compared with that of wild-type mice. Data was expressed as Mean ± SEM. (*n* = 6 each group, * *P* < 0.05, ** *P* < 0.01, *** *P* < 0.001 compared with the C57 dIoN-CCI group, two-way ANOVA with post-hoc Bonferroni test). dIoN-CCI, chronic constriction injury of distal infraorbital nerve; BL, baseline

Subsequently, we employed *Tlr2*^*−/−*^ mice to investigate the role of TLR2 on the development of persistent pain in the dIoN-CCI mouse model. The results showed that at 0.008 g mechanical stimulation intensity, *Tlr2*^*−/−*^ mice had a significant lower positive response rate on the ipsilateral side (F_time(5, 54)_ = 57.89, *P* < 0.0001; F_genotype(1, 54)_ = 112, *P* < 0.0001; F_time × genotype (5, 54)_ = 1.857, *P* = 0.1174; Fig. 5a) and contralateral side of dIoN-CCI surgery (F_time(5, 54)_ = 114.9, *P* < 0.0001; F_genotype (1, 54)_ = 149.1, *P* < 0.0001; F_time × genotype (5, 54)_ = 5.614, *P* = 0.0003; Fig. 5b) compared with wild-type mice. At 0.02 g stimulation intensity, *Tlr2*^*−/−*^ mice showed a significantly lower positive response rate on the ipsilateral side (F_time(5, 54)_ = 104.9, *P* < 0.0001; F_genotype (1, 54)_ = 136.6, *P* < 0.0001; F_time × genotype (5, 54)_ = 2.345, *P* = 0.0535; Fig. 5c) and contralateral side of dIoN-CCI surgery (F_time(5, 54)_ = 60.08, *P* < 0.0001; F_genotype (1, 54)_ = 64.59, *P* < 0.0001; F_time × genotype (5, 54)_ = 0.8623, *P* = 0.5124; Fig. 5d) compared with wild-type mice. Thus, these results suggested that TLR2 deletion alleviates dIoN-CCI induced bilateral mechanical pain hypersensitivity compared with that of wild-type mice.

### The inhibitory effects of BoNT/A treatment on the mRNA expression of glia activation markers and proinflammatory mediators in the TNC of the dIoN-CCI mice

**Fig. 6:**
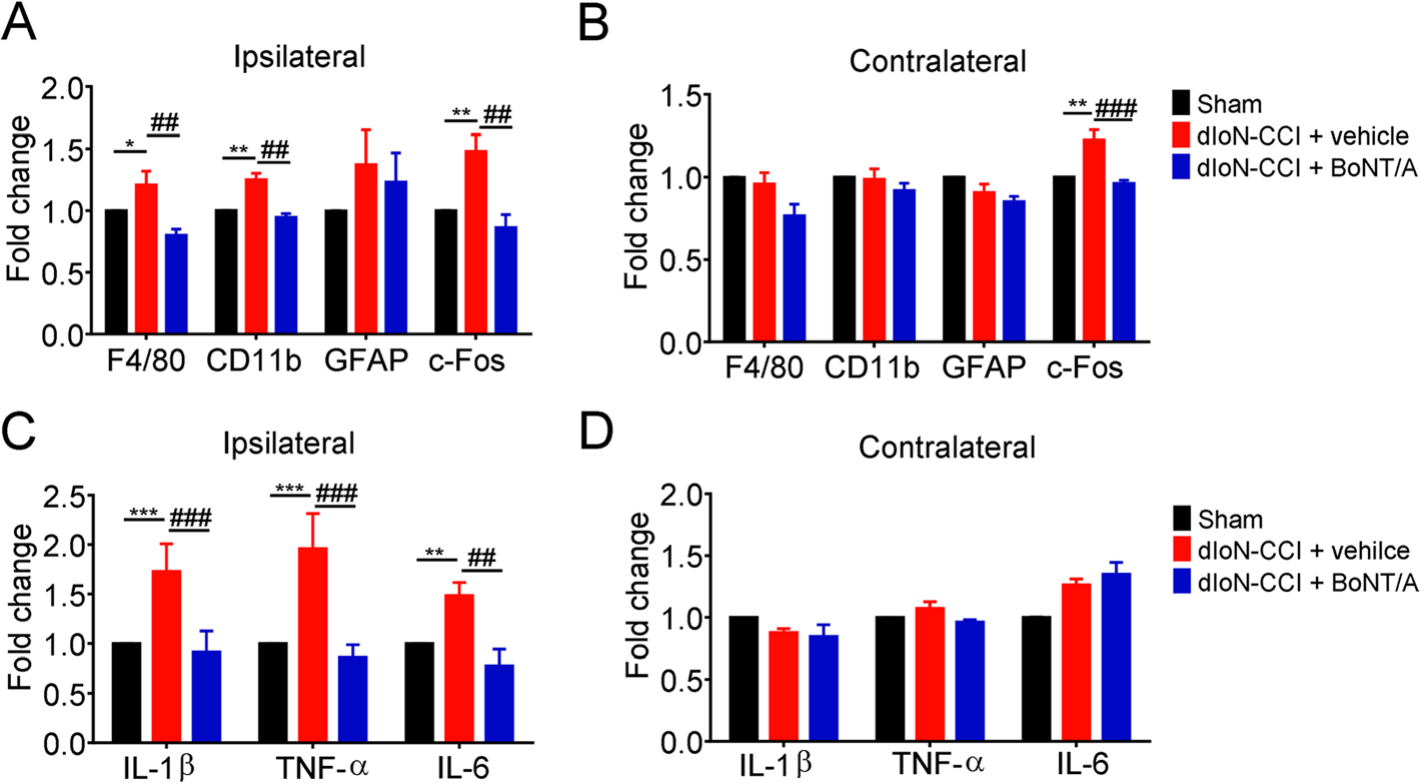
The expression of glia activation markers and proinflammatory factors in bilateral TNC site using RT-qPCR. **a** On the ipsilateral TNC of dIoN-CCI surgery, the mRNA expression of F4/80, CD11b and c-FOS were significantly increased by dIoN-CCI surgery, and BoNT/A treatment significantly decreased their up-regulation. **b** On the contralateral TNC of dIoN-CCI surgery, only the mRNA expression of c-FOS was increased by dIoN-CCI surgery, and BoNT/A treatment significantly decreased the up-regulation of c-FOS. **c** On the ipsilateral TNC of dIoN-CCI surgery, the mRNA expression of pro-inflammatory factors IL-1β, TNF-α and IL-6 were significantly elevated by dIoN-CCI surgery, and BoNT/A treatment significantly decreased their up-regulation. **d** There was no statistically significant difference in the expression of proinflammatory factors on the contralateral TNC of dIoN-CCI surgery. Data was expressed as Mean ± SEM. (*n* = 4 each group, ^*^ *P* < 0.05, ^**^ *P* < 0.01, ^***^ *P* < 0.001 compared with the sham group. ^**##**^ *P* < 0.01, ^**###**^ *P* < 0.001 compared with the dIoN-CCI group, unpaired Student’s t-test). TNC, Trigeminal nucleus caudalis; dIoN-CCI, Chronic constriction injury of distal infraorbital nerve; BoNT/A, Botulinum toxin A

We subsequently investigated the expression changes of glia and proinflammatory mediators in the TNC induced by TN and the possible effects of BoNT/A injection on these changes. On the ipsilateral side of dIoN-CCI surgery, the mRNA expression of markers F4/80 (macrophage or microglia marker), CD11b (microglia marker) and c-Fos (neuron activation marker) were significantly increased in the dIoN-CCI group compared with sham control (sham vs. dIoN-CCI + vehicle, F480: *t*_*6*_ = 2.965, *P* = 0.0351; CD11b: *t*_*6*_ = 0.3945, *P* = 0.0076; c-Fos: *t*_*6*_ = 4.707, *P* = 0.0033), and BoNT/A treatment significantly decrease these markers on day 5 after BoNT/A injection (day 19 after dIoN-CCI surgery) (dIoN-CCI + vehicle vs. dIoN-CCI + BoNT/A, F4/80: t_6_ = 4.799, *P* = 0.003; CD11b: t_6_ = 4.994, *P* = 0.0025; c-Fos: t_6_ = 5862, *P* = 0.0011; Fig. 6a). On the contralateral side of dIoN-CCI surgery, the mRNA expression of c-Fos was significantly increased in the dIoN-CCI group compared with sham group (sham vs. dIoN-CCI: t_6_ = 4.022, *P* = 0.0069), and the upregulation of mRNA expression of c-Fos was significantly decreased in BoNT/A-treated animals compared with that of vehicle-treated group (dIoN-CCI + vehicle vs. dIoN-CCI + BoNT/A, t_6_ = 8.647, *P* = 0.0001; Fig. 6b). The mRNA expression of proinflammatory factors IL-1β, TNF-α and IL-6 were significantly elevated in the ipsilateral side in dIoN-CCI surgery group compared with that of sham group (sham vs. dIoN-CCI + vehicle: IL-1β: t_6_ = 8.633, *P* = 0.0001; TNF-α: t_6_ = 8.990, *P* = 0.0001; IL-6: t_6_ = 5.629, *P* = 0.0013), and the upregulation of mRNA expression of these proinflammatory factors was significantly decreased on day 5 after BoNT/A injection (day 19 after dIoN-CCI surgery) animals (dIoN-CCI + vehicle vs. dIoN-CCI + BoNT/A, IL-1β: t_6_ = 10.39, *P* < 0.0001; TNF-α: t_6_ = 9.878, *P* < 0.0001; IL-6: t_6_ = 4.657, *P* = 0.0035; Fig. 6c). However, there was no statistically significant difference in the expression of proinflammatory factors in the contralateral side of dIoN-CCI surgery compared with that of sham animals (Fig. 6d). Together, the results suggested that dIoN-CCI surgery induced microglia activation and neuroinflammation in the TNC on the ipsilateral side of the surgery, and BoNT/A injection inhibited microglia activation and upregulation of proinflammatory factors in mice. Interestingly, BoNT/A treatment significantly reduced bilateral activation of neurons in the TNC induced by dIoN-CCI surgery, which may be associated with analgesic effects of BoNT/A on mirror image pain.

### Microglia activation in the ipsilateral TNC induced by dIoN-CCI surgery in mice

**Fig. 7:**
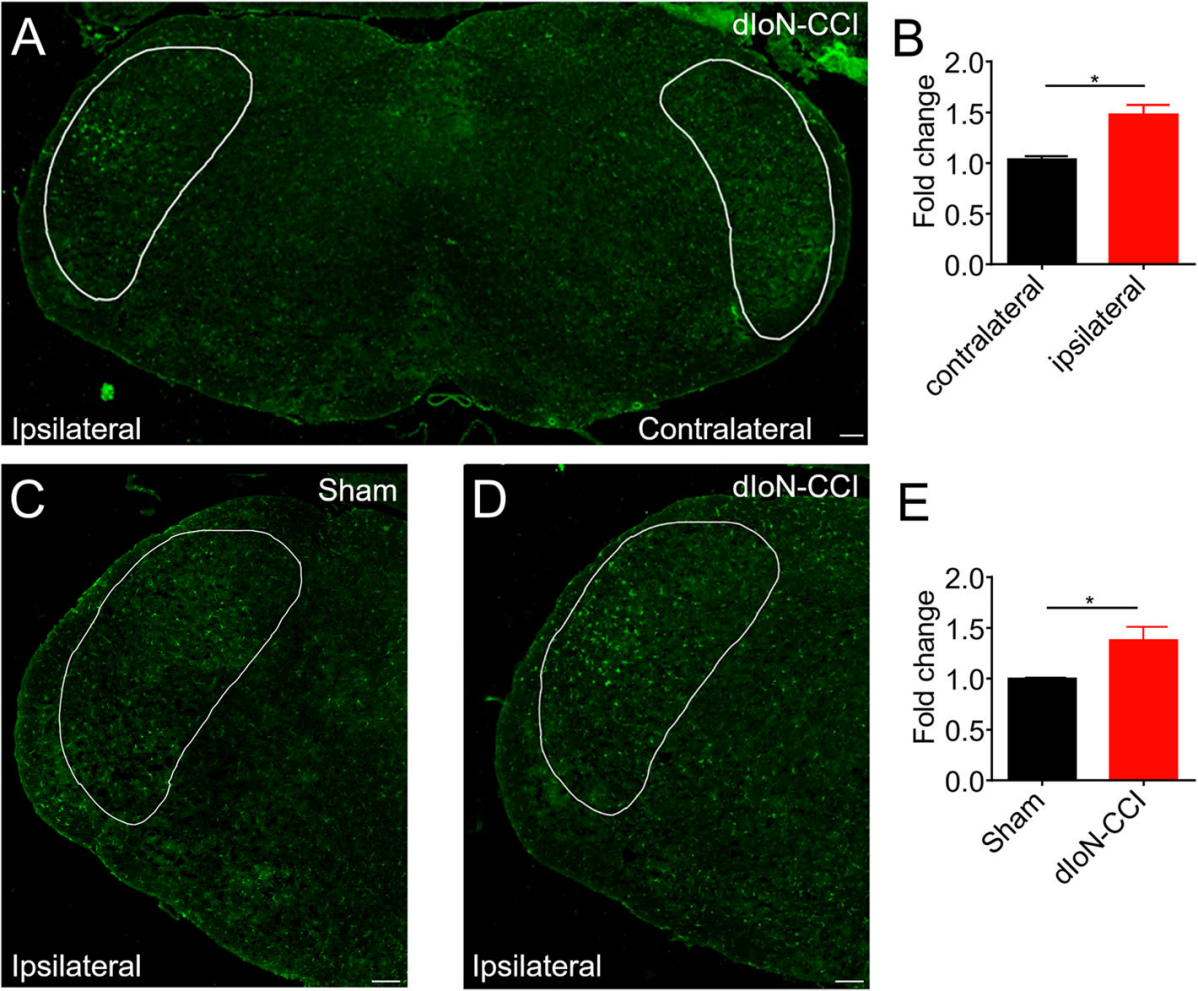
Immunostaining analysis showed the protein expression of IBA-1 in the TNC site (the area circled by the white solid circle). **a** Upregulation of IBA-1 expression in the ipsilateral TNC induced by dIoN-CCI surgery in mice. **b** Quantitative analysis of IBA-1 immunofluorescence intensity in the ipsilateral and contralateral TNC site in the dIoN-CCI group, as fold of contralateral side to surgery. **c** Expression of IBA-1 in the ipsilateral TNC in the sham mice. **d** Upregulation of IBA-1 expression in the ipsilateral TNC in the dIoN-CCI surgery mice. **e** Quantitative analysis of IBA-1 immunofluorescence intensity in the ipsilateral TNC between sham and dIoN-CCI group. Data was expressed as Mean ± SEM. (*n* = 3 each group, scale bar = 100 μm, ^*^ *P* < 0.05 compared with the control, unpaired Student’s t-test) TNC, trigeminal nucleus caudalis; dIoN-CCI, chronic constriction injury of distal infraorbital nerve

We then used IBA-1 (a microglia marker) immunofluorescence staining to investigate microglia activation in the TNC induced by dIoN-CCI surgery and the effects of BoNT/A on it. We showed that in on day 14 after dIoN-CCI surgery, there was upregulation of IBA-1-positive cells in the ipsilateral TNC of dIoN-CCI surgery, indicating ipsilateral activation in microglia induced by dIoN-CCI surgery in mice (Fig. 7a). There was no obvious changes of IBA-1 expression in the contralateral TNC of dIoN-CCI surgery group (Fig. 7a). Quantitative analysis of the intensity of IBA-1 immunofluorescence showed in the dIoN-CCI surgery mice, higher fluorescence intensity was found in the ipsilateral TNC than that of the contralateral side of the surgery (t_4_ = 4.332, *P* = 0.0123; Fig. 7b). By using immunostaining, we also compared the microglia activation in the ipsilateral TNC of surgery between the sham and dIoN-CCI surgery group. We found the IBA-1 fluorescence intensity in the ipsilateral side of TNC of dIoN-CCI mice was significantly higher than that of the sham group (t_4_ = 2.949, *P* = 0.0420; Fig. 7c-e). Thus, the results indicated that ipsilateral microglia activation in the TNC was induced by dIoN-CCI surgery compared with that of sham group.

### The inhibitory effects of BoNT/A treatment on upregulation of the expression of TLR2 and IBA-1 in the ipsilateral side of dIoN-CCI surgery in mice

**Fig. 8:**
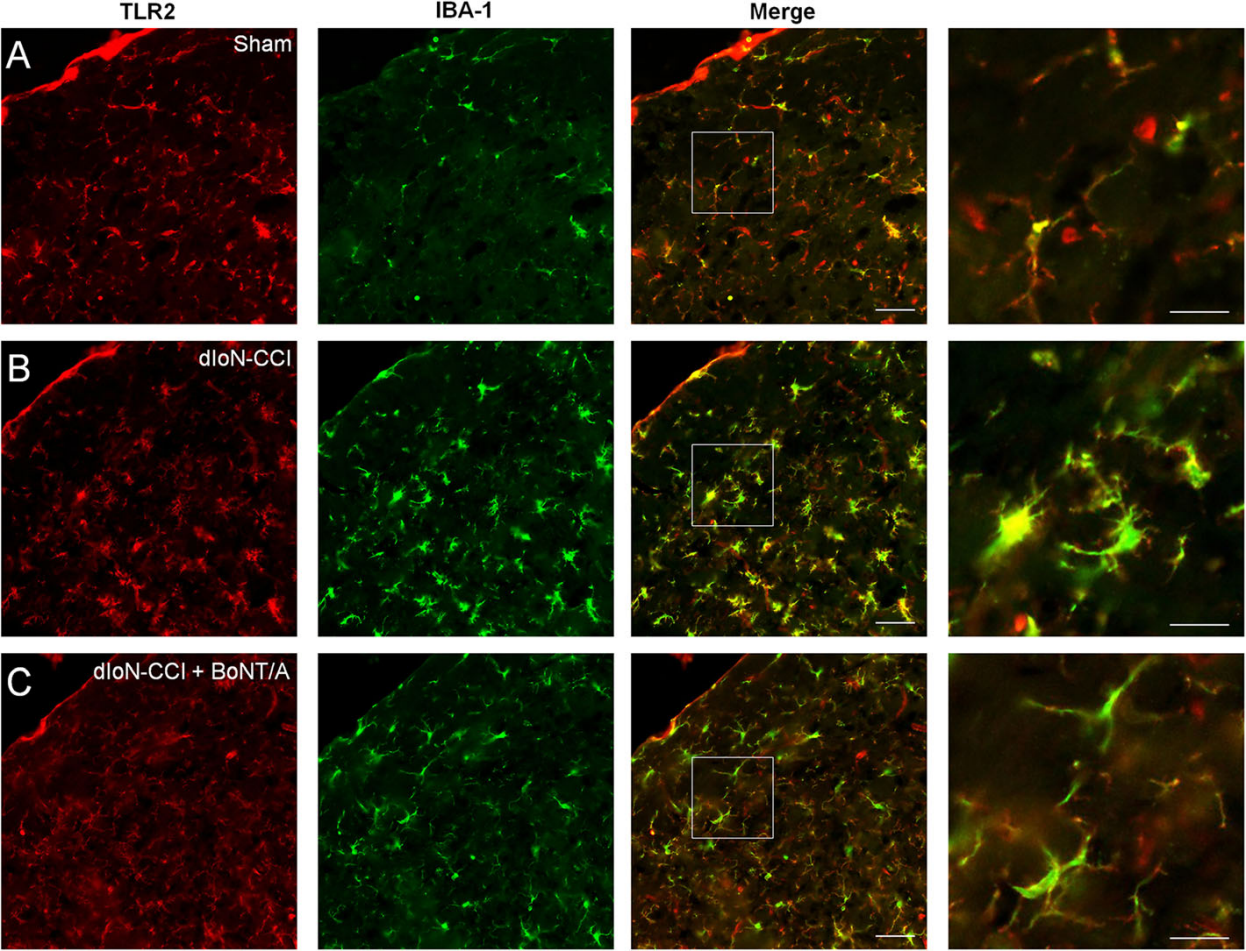
Double immunostaining of TLR2 and IBA-1 in the ipsilateral TNC induced by dIoN-CCI surgery in mice. **a** Double immunostaining of TLR2 and IBA-1 in the ipsilateral TNC induced in sham animals. **b** Double immunostaining of TLR2 and IBA-1 in the ipsilateral TNC in dIoN-CCI animals. **c** On the day 5 following BoNT/A treatment (on day 19 following dIoN-CCI surgery), double immunostaining of TLR2 and IBA-1 in the ipsilateral TNC in dIoN-CCI + BoNT/A animals. Scale bar = 50 μm, and the scale bar in the enlarged graph is 20 μm. TNC, Trigeminal nucleus caudalis; dIoN-CCI, chronic constriction injury of distal infraorbital nerve; BoNT/A, botulinum toxin A

By using immunofluorescence, the co-localization of IBA-1 and TLR2 at the TNC of the surgery side in all tested three groups was detected. Co-localization of IBA-1 and TLR2 was detected in the sham mice (Fig. 8a). On day 14 after dIoN-CCI surgery in mice, we found the increased expression of both TLR2 and IBA-1 in the activated microglia in the ipsilateral side of dIoN-CCI surgery (Fig. 8b). The upregulation of TLR2 and IBA-1 was significantly suppressed on day 5 after BoNT/A injection (day 19 after dIoN-CCI surgery) in dIoN-CCI mice (Fig. 8c). Thus, these results suggested that microglia activation was inhibited and co-localization of IBA-1 and TLR2 was reduced after BoNT/A injection in the TNC on the surgical side.

### BoNT/A reduced the upregulation of MyD88 expression after dIoN-CCI and TLR2 deficiency also reduced MyD88 expression

**Fig. 9:**
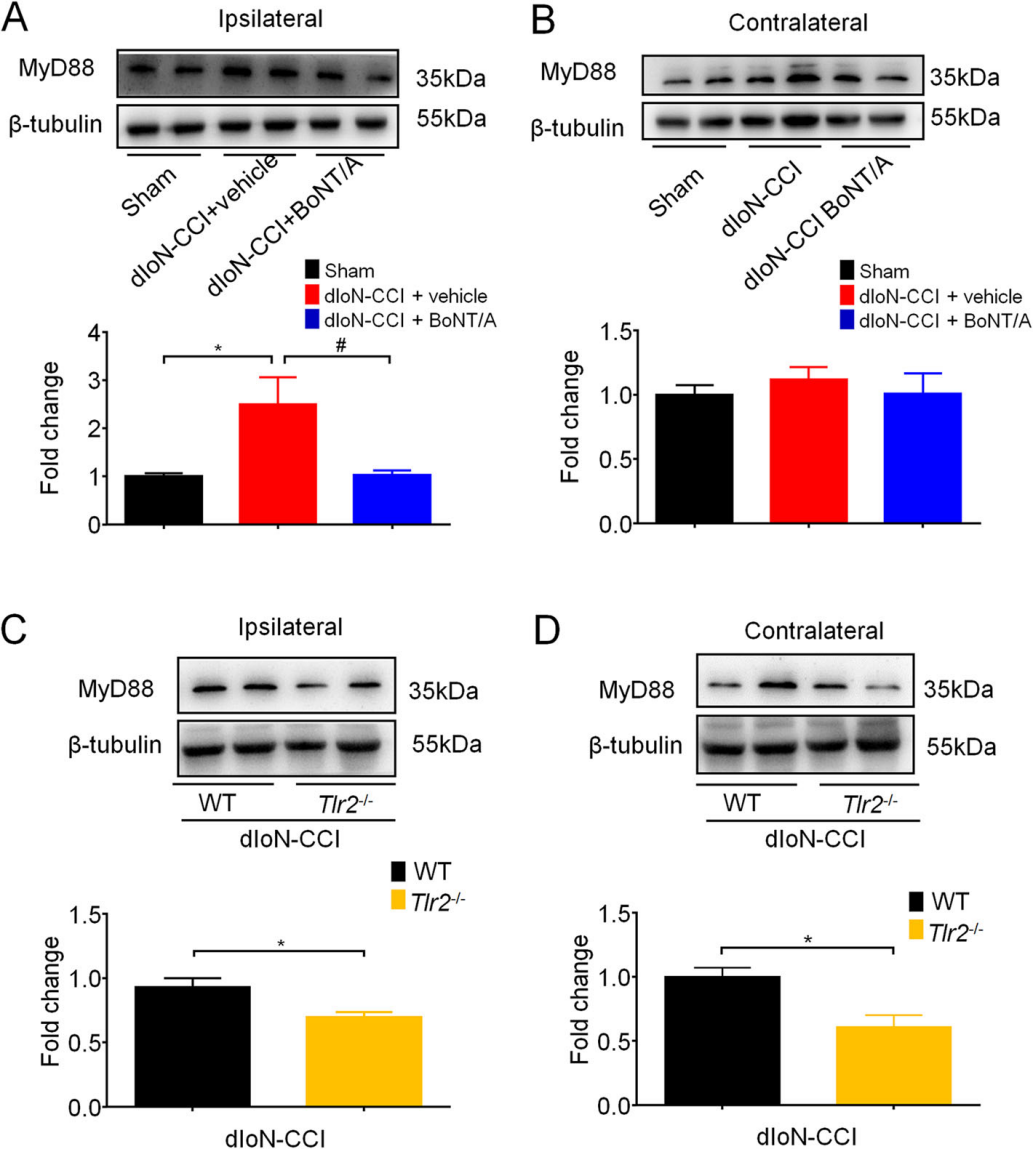
Western blotting analysis showed the expression changes of Myd88 in the bilateral TNC in mice. **a** The expression of MyD88 was increased by dIoN-CCI surgery compared with the sham group, and BoNT/A injection decreased this upregulation. **b** The expression of MyD88 did not change in the contralateral TNC by dIoN-CCI surgery compared with the sham group. **c**-**d** The expression of MyD88 in the ipsilateral and contralateral TNC was significantly reduced in *Tlr2*^−/−^ mice compared with wild-type mice following dIoN-CCI surgery. Data was expressed as Mean ± SEM. (*n* = 4 each group, ^*^ *P* < 0.05 compared with the sham group. ^**#**^ *P* < 0.05 compared with the TN group, unpaired Student’s t-test) TNC, Trigeminal nucleus caudalis; dIoN-CCI, Chronic constriction injury of distal infraorbital nerve; BoNT/A, Botulinum toxin A

Finally, western blotting analysis were used to measure the protein expression level of MyD88 in the TNC in all tested three groups. The results showed that the expression level of MyD88 on the ipsilateral TNC of the surgery was significantly elevated in the dIoN-CCI surgery mice compared with that of sham mice (sham vs. dIoN-CCI + vehicle, t_6_ = 2.718, *P* = 0.0347), and upregulation of the expression of MyD88 was significantly decreased on day 5 after BoNT/A injection (day 19 after dIoN-CCI surgery) in mice (dIoN-CCI + vehicle vs. dIoN-CCI + BoNT/A, t_6_ = 2.654, *P* = 0.0378; Fig. 9a). However, the expression of MyD88 did not change in the contralateral TNC of the surgery (sham vs. dIoN-CCI, t_6_ = 0.9386, *P* = 0.3842; dIoN-CCI vs. dIoN-CCI + BoNT/A, t_6_ = 0.5799, *P* = 0.5831; Fig. 9b). On day 14 after dIoN-CCI surgery in mice, the expression of MyD88 in the bilateral TNC of *Tlr2*^*−/−*^ mice was significantly reduced compared with that of wild-type mice (ipsilateral: t_4_ = 2.923, *P* = 0.0431; Fig. 9c; contralateral: t_4_ = 3.438; *P* = 0.0138; Fig. 9d). Thus, these data indicated that MyD88 as downstream molecule of TLR signaling is involved in the pathogenesis of TN and BoNT/A injection could reduce the expression level of MyD88 in the ipsilateral TNC.

## Discussion

In this study, we found that peripheral injury of the trigeminal nerve following dIoN-CCI surgery induced trigeminal neuropathic pain and anxiety-like behavior in mice, triggering microglia activation in the ipsilateral TNC, with the up-regulation of microglia TLR2 expression in the TNC. Importantly, peripheral s.c. injection of BoNT/A significantly attenuated bilateral mechanical pain hypersensitivity and anxiety-like behavior induced by dIoN-CCI surgery, which was associated with the inhibition of the microglia activation, decreased expression of TLR2, and reduced expression of several proinflammatory mediators in the TNC in mice.

The pathogenesis of primary TN are still largely unclear. To date, there are mainly three hypotheses to explain it. The first is the trigeminal nerve compression theory. The abnormal neurovascular compression of the trigeminal nerve at the pons stimulates the trigeminal nerve root, thus producing pain. Jannetta treated the patient of TN with microvascular decompression surgery, and pain disappeared after surgery, strongly supporting this theory [21]. The second is the central sensitization theory. The outbreak of TN have the characteristics of epileptic attack, and it is believed that pain may be associated with thalamic-cortico-trigeminal spinal tract nucleus irritative lesions [22]. The use of antiepileptic drugs in the treatment of TN may support this hypothesis [22]. The third is the neurodegeneration theory. The biopsy of the dysfunctional trigeminal nerve was found to produce demyelination changes and a “short-circuit” between two adjacent fibers of the demyelinated nerve may cause abnormal nerve transmission, leading to pain responding to a slight stimulus [23].

The previous clinical trials showed that BoNT/A was useful for the relief of TN in patients [24]. It was found that subcutaneous injections of BoNT/A produced analgesia lasting 60 days in 13 patients with TN, who reduced or discontinued prophylactic medication and switched from multi-drug therapy to monotherapy without adverse effects [23]. Similarly, the previous study found that BoNT/A therapy was effective and safe in the treatment of elderly (≥80 years) TN patients at doses comparable to their more youthful counterparts (< 60 years) [25]. Our present animal experiment confirmed that unilateral facial injection of BoNT/A attenuated bilateral mechanical pain hypersensitivity in a mouse TN model, which was induced by performing dIoN-CCI surgery in mice. Thus, our data indicated that local injection of BoNT/A may be effective for widespread pain in patients, including mirror image pain.

Epidemiological studies have shown that psychiatric disorders (such as anxiety) are common in patients with chronic pain [26–28]. Previous most studies on pain and anxiety have typically been conducted separately [29]. Chronic pain and anxiety are common comorbidities, and they could promote their development mutually [30, 31]. In the present study, we found that facial injection of BoNT/A could remarkable inhibit anxiety-like behavior in dIoN-CCI surgery mice. Our previous work showed that single facial injection of BoNT/A could have anti-depressant effects in depression model induced by chronic stress in mice [8]. Additionally, we also tested depression-like behavior in dIoN-CCI mouse model by using forced swimming test. However, we did not find significant difference in forced swimming test between sham and dIoN-CCI surgery mice. Nevertheless, BoNT/A treatment attenuated both trigeminal neuropathic pain and accompanying anxiety-like behavior in mice, indicating BoNT/A treatment may be useful for the management of chronic pain-induced anxiety.

In the central nervous system, gliosis in response to injury often involves the proliferation or hypertrophy of glial cells [32]. Microgliosis exhibits profound morphological changes from branchial microglia to amoeboid microglia with enlarged cell bodies. In 1993, Erickson and his colleagues demonstrated significant microgliosis in the spinal cord and brainstem regions after nerve injury, by histochemical staining of OX-42 (antibodies recognizing CD11b) [33]. This is consistent with our findings on the morphological changes of microglia in the ipsilateral TNC induced by dIoN-CCI surgery in mice. The specific role of microglia in pain was discovered in 2003, when three independent studies showed the involvement of microglia in neuropathic pain after peripheral nerve injury [34–36]. There is growing evidence that microglia drive central sensitization through microglial mediators. Microglia are a major source of cytokines, including TNF-α and IL-1β [37]. The reduction or loss of inhibitory synaptic transmission in the pain circuitry of the spinal cord dorsal horn is also closely associated with central sensitization [38]. Notably, microglial mediators such as cytokines, PGE2 and BDNF, regulate inhibitory synaptic transmission in spinal cord dorsal horn neurons through presynaptic, postsynaptic and/or extrasynaptic mechanisms [37]. Pro-inflammatory cytokines strongly regulate inhibitory synaptic transmission in multiple sites in the dorsal horn of the spinal cord [39]. At the presynaptic level, IL-1 and IL-6 inhibit the frequency of spontaneous inhibitory postsynaptic currents (sIPSCs) in spinal pain circuits. At postsynaptic sites, IL-1 and IL-6 decreased the amplitude of sIPSCs. At extrasynaptic sites, GABA and glycine receptor activity could be inhibited by IL-1β [40, 41]. In addition, TNF-α rapidly inhibited the spontaneous action potentials of GABAergic neurons [42]. Zychowska et al. determine the effects of BoNT/A on pain-related behavior and the levels of glial markers and interleukins in the spinal cord and dorsal root ganglia (DRG) after chronic constriction injury (CCI) to the sciatic nerve in rats. It was found that injection of BoNT/A suppressed the CCI-induced upregulation of IL-18 and IL-1β in the spinal cord and/or DRG and increased the levels of IL-10 and IL-1RA in the DRG. They suggest that BoNT/A significantly attenuates pain-related behavior and microglial activation and restores the neuroimmune balance by decreasing the levels of pro-nociceptive factors (IL-1β and IL-18) and increasing the levels of anti-nociceptive factors (IL-10 and IL-1RA) in the spinal cord and DRG [43]. In the present study, our RT-qPCR and immunostaining data demonstrated that BoNT/A treatment significantly inhibited microglia activation in the ipsilateral TNC induced by dIoN-CCI surgery in mice. In addition, our results showed that the mRNA expression of IL-1β, TNF-α and IL-6 in the ipsilateral TNC was also upregulated following dIoN-CCI surgery in mice, and BoNT/A treatment significantly inhibited the upregulation of these proinflammatory factors in the ipsilateral TNC. Thus, our findings indicated that peripheral administration of BoNT/A was able to suppress microglia-mediated neuroinflammation in the central nerve system.

BoNT/A therapy is a very useful strategy for the treatment of neuropathic pain, but how it induces functional recovery remains unknown. Neurohistology analysis confirmed that the distal part of the regenerative axon myelination had high expression of S100 after BoNT/A injection (S100 is used as a Schwann cell marker). Low doses of BoNT/A were not adequate to produce muscle dysfunction, instead, sensory-motor recovery was accelerated by stimulating the regeneration of axon myelination [44]. However, whether injection of BoNT/A promotes myelin regeneration or not warrants further investigation [45].

MyD88 is a key downstream adapter for most TLRs and IL-1Rs. Deletion of MyD88 in mice results in susceptibility to various pathogens [46]. Previous studies have shown that deletion of TLR4 can attenuate neuropathic pain in mice [47, 48]. However, the role of TLR4 in the pathogenesis of TN was not investigated yet. After TN, the deletion of TLR4 did not reduce the expression of MyD88, a downstream molecule of TLRs [49]. A recent study on TN showed that TLR8 activate ERK and p38, and further increase the expression of pro-inflammatory cytokines in the trigeminal ganglion neurons [50]. In our study, MyD88, which is an adaptor for all TLRs (except TLR3). dIoN-CCI surgery elevate the mRNA expression of TLR2 and TLR5 in the ipsilateral TNC in mice. After screening different TLRs expressed in the TNC, we focused our study on the role of TLR2 in the pathogenesis of TN [51], although there are no previous studies on the role of TLR2 in the pathogenesis of TN. Our study showed that TLR2 and MyD88 expression was upregulated in the TNC after dIoN-CCI surgery in mice. We established the same TN model on *Tlr2*^*−/−*^ mice and wild-type mice to test mechanical pain and took the TNC tissue for western blotting. It was found that the bilateral mechanical pain hypersensitivity was significantly reduced and the upregulation of MyD88 expression was also decreased significantly in *Tlr2*^*−/−*^ mice. Interestingly, BoNT/A treatment was shown to reduce the mRNA and protein expression of TLR2 and its downstream MyD88 in the ipsilateral TNC in TN mouse model. Thereby, BoNT/A treatment attenuated trigeminal neuropathic pain, which may be associated the inhibition of TLR2-mediated neuroinflammation in the TNC.

Finally, our findings suggest that unilateral ligation of branches of the trigeminal nerve can cause bilateral trigeminal nociceptive hyperalgesia in mice, which is also known as mirror image pain (Fig. 1). RT-qPCR assay also showed neuronal activation by elevated expression of c-Fos in the contralateral TNC following dIoN-CCI surgery. Injuries to peripheral nerves or spinal roots produce a number of breakdown products at the lesion and beyond, with a subsequent response from immunocompetent cells [52]. This inflammatory response releases a number of immunomodulatory cytokines that can be transported through the blood or cerebrospinal fluid (CSF) to contralateral sites in the body and affect the spinal roots or peripheral nerves [53, 54]. Neurons are elements of a highly organized neuronal network, and injury to peripheral neurons results in signals entering the central nervous system via neurons, which then eventually affect the contralateral ipsilateral neurons [55, 56]. Activation of glial cells is also thought to be involved in the phenomenon of ipsilateral or contralateral spread of pain [57]. The nerve-glial interaction is bidirectional. On the one hand, glial cells express different types of neurotransmitter receptors, which allow them to respond to neural signals [58]. On the other hand, glial cells produce many neuroactive mediators (e.g. pro-inflammatory cytokines and growth factors) [59]. Studies have shown that after peripheral injury, satellite glia in the contralateral dorsal root ganglion are activated by TNF-α, which diffuses through the CSF from the injured side. Activated satellite glia produce additional nerve growth factor to enhance nociceptive excitability, inducing the pain of contralateral side [5]. We hypothesize that the relief of TN on the contralateral side of surgery after BoNT/A injection is due to the reduction of TNF-α and other inflammatory factors released from the TNC site on the side of surgery. Then the diffusion of inflammatory factors through the cerebrospinal fluid is not sufficient to activate the contralateral satellite glia to release NGF, so the contralateral central neurons remain stationary. This is what we need to prove in the future.

Of note, there are several limitations of our study. First, we only investigated the therapeutic mechanism of BoNT/A for trigeminal neuralgia in male mice. Given more and more studies employed both male and female animals, it remains to be investigated whether BoNT/A exerts similar therapeutic effects in female animals. Whether inhibition of microglia contributes to the therapeutic effects of BoNT/A in both male and female animal warrants further investigation. Second, there are several other TN animal models besides dIoN-CCI surgery developed in mice. Therapeutic effects of BoNT/A should be confirmed by using different TN animal models. Third, it was noticed after injection of BoNT/A, flaccid paralysis of the facial muscles at the injection site was observed. The side effects of BoNT/A therapy for TN should be investigated in the future. Finally, the direct inhibitory effects of BoNT/A on microglia and the underlying mechanisms in vitro should be explored.

## Conclusion

In conclusion, our study showed that TN caused the activation of microglia, upregulation of TLR2 and MyD88 expression, causing elevated expression of inflammatory factors in the ipsilateral TNC site following dIoN-CCI surgery in mice. Peripheral unilateral injection of BoNT/A could inhibit bilateral trigeminal neuropathic pain and anxiety-like behaviors, which is associated with the inhibition of microglia activation, the upregulation of expression of TLR2, its downstream of MyD88 and proinflammatory factors in the ipsilateral TNC and inhibit the activation of neurons in the contralateral TNC following dIoN-CCI surgery in mice. Therefore, we have provided preclinical evidence for the effectiveness of BoNT/A therapy on the management of TN and its comorbidity anxiety.

## Data Availability

The datasets used and/or analyzed during the current study are available from the corresponding author on reasonable request.

## Abbreviations

TN: Trigeminal neuralgia
BoNT/A: Botulinum toxin type A
MAPK: Mitogen-activated protein kinases
TLRs: Toll-like receptors
TLR2: Toll-like receptor 2
Myd88: Myeloid differentiation factor
GFAP: Glial fibrillary acidic protein
IBA-1: Ion calcium-binding bridging molecule 1
TNF-α: Tumour Necrosis Factor alpha
IL-1β: Interleukin 1 beta
IL-6: Interleukin-6
CCI: Chronic constriction injury
IoN: Infraorbital nerve
TG: Trigeminal ganglion
TNC: Trigeminal nucleus caudalis
